# Can Sanitary Surveys Replace Water Quality Testing? Evidence from Kisii, Kenya

**DOI:** 10.3390/ijerph14020152

**Published:** 2017-02-07

**Authors:** Aaron Gichaba Misati, George Ogendi, Rachel Peletz, Ranjiv Khush, Emily Kumpel

**Affiliations:** 1 Department of Environmental Science, Egerton University, Njoro Campus, P.O. Box 536, Egerton 20115, Kenya; gichabaron@gmail.com (A.G.M.); gmorara2009@gmail.com (G.O.); 2 The Aquaya Institute, Nairobi 00505, Kenya; rachel@aquaya.org; 3 The Aquaya Institute, Larkspur, CA 94939, USA; ranjiv@aquaya.org

**Keywords:** rural water supply, sanitary surveys, water quality, springs, dug wells, rainwater harvesting

## Abstract

Information about the quality of rural drinking water sources can be used to manage their safety and mitigate risks to health. Sanitary surveys, which are observational checklists to assess hazards present at water sources, are simpler to conduct than microbial tests. We assessed whether sanitary survey results were associated with measured indicator bacteria levels in rural drinking water sources in Kisii Central, Kenya. Overall, thermotolerant coliform (TTC) levels were high: all of the samples from the 20 tested dug wells, almost all (95%) of the samples from the 25 tested springs, and 61% of the samples from the 16 tested rainwater harvesting systems were contaminated with TTC. There were no significant associations between TTC levels and overall sanitary survey scores or their individual components. Contamination by TTC was associated with source type (dug wells and springs were more contaminated than rainwater systems). While sanitary surveys cannot be substituted for microbial water quality results in this context, they could be used to identify potential hazards and contribute to a comprehensive risk management approach.

## 1. Introduction

In sub-Saharan Africa, unsafe drinking water was estimated to cause more than 200,000 deaths in 2012 [1]. Information about water quality can be used to manage the safety of water sources. While regulatory structures often exist that specify the responsibilities of health or water surveillance agencies to test water sources, institutions in this region often have limited capacity to carry out these responsibilities [2,3,4]. Water quality testing is expensive and complicated: collecting and testing water samples requires labor, equipment, consumables, transportation, and training [5,6,7].

Given the large amount of resources required for testing water quality, there is a need to improve its cost-effectiveness. Alternative approaches for identifying high-risk water supplies such as sanitary surveys, which are observational checklists for identifying potential hazards, have been promoted by environmental health specialists and the World Health Organization (WHO) [8]. Sanitary surveys can be completed quickly and require no special equipment, making them less expensive and easier to implement than microbiological testing [6,8]. However, previous research on whether risks assessed through sanitary surveys correspond to measured water quality has produced mixed results and focused primarily on solely groundwater sources. For example, a study in Amuria District, Uganda, found that the water source type was a better predictor of fecal contamination than sanitary scores (the sum of hazards present in the sanitary survey); boreholes had the best microbiological water quality, followed by open dug wells, protected springs, and, finally, surface water, but there was only a weak correlation between thermotolerant coliform (TTC) concentrations and variations in sanitary scores [9]. A study in County Cork, Ireland, found no correlation between sanitary survey–identified hazards and TTC, although they noted there were too few contaminated samples to make an adequate comparison [10]. In contrast, a study in Dar es Salaam, Tanzania, found that the sanitary score predicted up to 87% of the measured *Escherichia coli* concentrations among wells [6]. Similarly, a study of shallow protected springs in Kampala, Uganda, identified a positive relationship between the sanitary score and microbial water quality, although no significant correlation was identified when data from low- and high-density population areas were analyzed separately [11]. It is therefore likely that correlations between microbiological indicator organism measurements and sanitary scores are water source– and context-specific; more research is needed to understand when it is appropriate to use sanitary surveys as a supplement or replacements for water quality testing.

Our objective was to assess whether the results of sanitary survey inspections were associated with measured indicator bacteria levels in rural drinking water sources. We focused on rural areas of Kisii Central, a sub-county in Kenya. In Kenya, while 43% of households used improved water sources in 2015, these improved sources may not provide water that is safe. For example, in Kisii County, Kenya, the population without access to safe drinking water was estimated to be at least nine percentage points higher when improved sources that contained microbial contamination were excluded from the proxy definition of ‘safe’. While water quality testing is performed by public health surveillance agencies in Kisii County, there are more water sources than can be reasonably tested within the three- to five-year recommended time frame [8]. The results of this study can suggest whether sanitary surveys can be used to complement or replace water quality testing to inform water safety management activities. We also evaluated the variability in fecal contamination between two samples collected from a point water source to assess the information gained or lost from one-time sampling of a source.

## 2. Materials and Methods

### 2.1. Study Site

**Figure 1:**
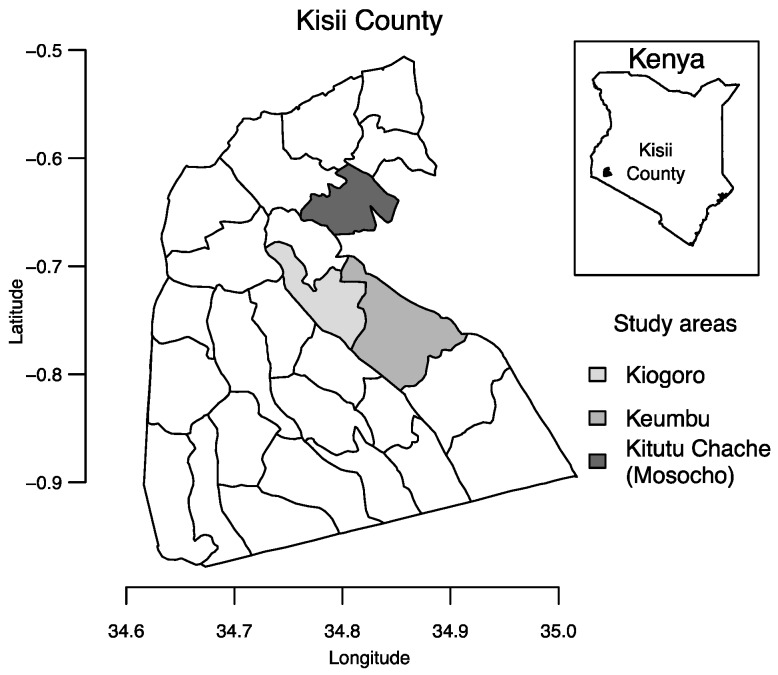
Kisii County with the study areas of Keumbu, Kiogoro, and Kitutu Chache (which includes Mosocho as a smaller area within Kitutu Chache). Shapefiles were obtained from DIVA-GIS (diva-gis.org).

Kisii Central is an administrative division of Kisii County, Kenya, which is located southeast of Lake Victoria with a population of 1.1 million people [17] (Figure 1). Average annual rainfall in Kisii is estimated at 1500 mm [18].

In 2011, protected springs were the most frequently used water sources in rural areas of Kisii County (used by 55.9% of the population), followed by unprotected springs (used by 27.2% of the rural population) and surface water (used by 7.6% of the rural population) [19]. Rainwater collection systems were used by 3.6% of the rural population, protected dug wells and unprotected dug wells were used by 2.1% of the rural population each, and less than 2% of the remaining population used piped water or other sources. Aquifers in the Gucha catchment, of which most of Kisii County is included, range from depths of 13–60 m [20].

### 2.2. Study Design

We used stratified sampling to select 61 drinking water sources to sample based on an inventory of drinking water sources obtained from the Kisii County District Public Health Office (KCDPHO) [3]. In February 2014, KCDPHO Community Health Workers (CHW) had worked with village elders to count all water sources in each village to create the inventory. The total number of selected sources was based on available resources for testing. We selected the number of sources to sample in each rural administrative division (Mosocho, Keumbu, and Kiogoro) roughly in proportion to the total number of water sources in the inventory in each division (Figure 1). The inventory listed 7400 sources: 3981 in Mosocho (6% springs, 33% dug wells, and 61% rainwater harvesting systems), 2188 at Keumbu (8% springs, 4% wells, and 88% rainwater harvesting systems), and 1231 at Kiogoro (5% springs, 1% wells, and 92% rainwater harvesting systems). Sources accounting for <1% of the inventory included boreholes, rivers, rock catchments, and piped schemes. We sampled 32 water sources from Mosocho, 21 from Keumbu, and eight from Kiogoro.

To select water sources within each division for sampling, we consulted with KCDPHO CHWs who recommended the water sources in each division that were most commonly used by community members. Although rainwater harvesting systems (RWH) accounted for the majority of sources in all divisions, many of these RWH were used only by individual households, while springs were shared by villages and dug wells were frequently shared by multiple households. Therefore, a higher proportion of the population used springs or dug wells, as indicated by the census data. Therefore, we selected 25 springs, 20 wells, and 16 RWH for sampling. Since we prioritized sources used by a large number of people, the sampled springs were shared by communities (the local government was involved in their protection, however, there were no clear structures for managing the springs), the sampled wells were commonly built, shared, and managed by several households, and sampled RWH were constructed and managed by institutions (e.g., schools, hospitals).

We sampled selected water sources twice within one month, one week apart, to capture variability in water quality, in August 2014. The two samples from the same source were collected between seven to 13 days apart.

### 2.3. Sample Collection and Analysis

Samples from springs were directly collected from the spring outlet. Samples from wells were collected by drawing from a sterilized bucket that had been wiped with ethanol and rinsed with deionized water. Taps at rainwater tanks were sterilized with a flame before sample collection. Water samples were collected in 100 mL Whirl-Pak collection bags (Nasco, Modesto, CA, USA) and put on ice for transport to a laboratory located at the Gusii Water and Sanitation Company in the town of Kisii and tested within 6 h.

We quantified TTC counts using the membrane filtration system included with the Potakit^®^ test kit (Palintest, Ltd., Gateshead, UK). We tested sample blanks daily. We measured temperature, pH, electrical conductivity (EC), and turbidity on site using probes in the Potakit test kit. Calibrations for the equipment were performed before fieldwork began.

### 2.4. Sanitary Survey

We also conducted a sanitary survey for each selected water source. The sanitary survey questionnaire relied on a list of observations, unique for each water source type (springs, dug wells, and RWH). We used the sanitary surveys in the WHO Guidelines for Drinking Water Quality (GDWQ) and modified these questionnaires during piloting to include additional risk factors recommended by CHWs and exclude questions not applicable to the study area (Table 1) [8].

At each water source, questions in the sanitary survey that identified a water quality risk were given a score of one point while those that did not identify a risk were given a score of zero points. We totaled the points for each water source and divided by the total number of questions answered to arrive at a risk score (RS) for each source. We then grouped the RS into low (0%–30%), medium (40%–50%), high (60%–70%), and very high (80%–100%) risk categories [6]. We also recorded whether it had rained the day before sampling.

### 2.5. Data Analysis

Data were entered into Excel and analyzed with Excel and R software (R Core Team 2015, Vienna, Austria). TTC data were log transformed and used a value of log_10_(*x*+1). We used a non-parametric test, the Wilcoxon rank sum test, to determine the significance of differences in the measured water quality parameters from water sources with different sanitary features. Samples were classified as TNTC if the TTC count exceeded 300 CFU/100 mL. Conductivity was converted to total dissolved solids (TDS) by multiplying the default value in the meter of 0.5 by the EC values. The significance level was set at *p* < 0.05.

## 3. Results

### 3.1. Water Quality by Water Source Types

A total of 61 water sources (25 springs, 20 dug wells, and 16 RWH) were sampled, with 45 sources tested twice (n = 106 samples). Contamination by TTC was frequent in all sources: 87% of all samples (n = 106) contained TTC, with a median of 107 CFU/100 mL (Table 2). All samples from dug wells contained TTC, while most of the water samples from springs (95%, n = 41) and RWH (61%, n = 31) tested positive for TTC (Table 2). The levels of TTC in RWH were significantly lower than in the other source types (*p* ≤ 0.01, Wilcox rank sum) (Table 2).

Dug wells had the highest mean concentration of TDS (46 mg/L), followed by springs (44 mg/L) and RWH (6 mg/L), although all were below the WHO recommended value of 500 mg/L (Table 2). Turbidity varied in different water source types, with the mean turbidity in dug wells (11.6 NTU) and springs (6.7 NTU) above the WHO recommended value of 5 NTU for small systems, while the mean turbidity in RWH of 3.8 NTU was below this value [4]. pH and temperature were consistent across water sources, with averages of 5.8 and 23.0 °C, respectively (Table 2).

### 3.2. Sanitary Risk Factors

Dug wells carried the highest mean RS, with a score of 58% across all wells, followed by springs (45%) and rainwater harvesting systems (32%) (Table 3). However, no single risk factor for any source type was associated with significantly higher TTC levels (Table 3, Wilcox rank sum).

#### 3.2.1. Dug Wells

Hazards were frequently observed at dug wells, and half (50%) were unprotected by masonry. No wells were fenced, almost all had unsealed walls (95%), most open wells had a rope and bucket that were vulnerable to contamination (84%), and most had a latrine nearby (80%). More than half of the wells also had pollution nearby (including fertilizer, crude dumping of waste, and runoffs) and an inadequate parapet (70% each). In addition, most lacked a cover, had a nearby latrine at a higher elevation, had stagnant water nearby, or were vulnerable to flooding (60% each). All wells had an adequate floor that exceeded 1 m with few cracks (10%), and clothes washing and open defecation were observed near only one well. Animals grazed nearby 40% of the dug wells (Table 3).

#### 3.2.2. Springs

Forty-four percent of springs were unprotected (lacking a brick, masonry, or concrete ‘spring box’ around the spring to direct water flow to storage or distribution) (Table 3). The most common hazards identified among the sampled springs were lack of a surface water diversion ditch (96%), lack of a fence (88%), and human activity near the spring (84%). Relatedly, animals could reach within 10 m of almost half of the observed springs (48%), and washing of clothes was observed at a third of the springs (36%). Latrines were observed uphill at 40% of the springs. The least frequently observed hazards included open defecation (observed at one spring) and grazing animals (observed at two springs) (Table 3).

#### 3.2.3. Rainwater Harvesting Systems

There were few potential hazards identified for RWH. Three-quarters lacked a cover (75%), and more than half (62%) had visible contamination of the roof catchment area or a tank opening vulnerable to intrusion (56%). However, other risk factors were seldom observed: no sources of pollution were observed around the water collection area, all roofs were tarred, and only one had dirty gutters (Table 3).

### 3.3. Composite Risk Scores

**Figure 2:**
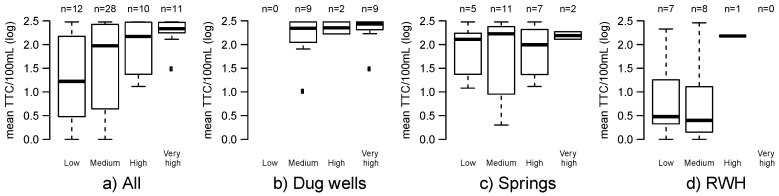
Boxplots of the thermotolerant coliform (TTC) concentrations for each RS category by: (**a**) All water sources; (**b**) Dug wells; (**c**) Springs; (**d**) Rainwater harvesting (RWH) systems.

While the overall RS showed a trend of increasing RS and increasing concentrations of TTC among all source types (Figure 2a), this was largely driven by source type (Figure 2b–d). All dug wells were categorized at or above the medium risk category (Figure 2b), and springs had RS values ranging from low to very high (Figure 2c). RWH had the lowest RS values of any water source type and none were categorized as ‘very high’ risk, although there were fewer questions for RWH than for other sources (Table 1, Figure 2d). There was no trend between increasing RS and TTC levels when data were disaggregated by individual source type (Figure 2b–d).

### 3.4. Variability within Sources

**Figure 3:**
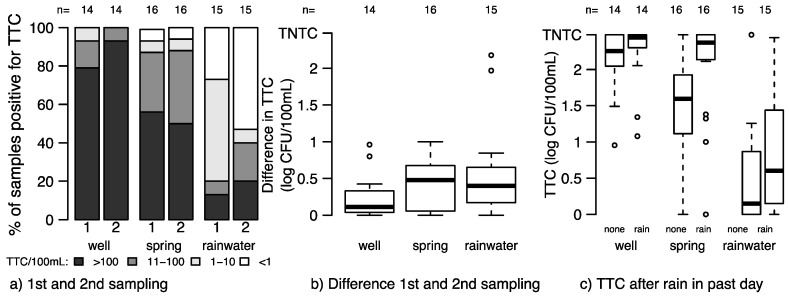
Variability between results from two samples collected from the same water source. (**a**) Percent of samples positive for TTC during the first and second samplings among sources tested twice; (**b**) Boxplot of the difference in TTC between the first and second sampling in each source type among the sources tested twice; (**c**) Boxplot of the TTC concentrations in samples collected when no prior rain (none) or rain in the last day (rain) in all tested source types. TNTC: too numerous to count.

Forty-five of the water sources were tested twice to assess variability between different sampling times. In aggregate, the percentages of samples positive for TTC were the same for both dug wells and springs between the first and second samplings, but fewer samples from RWH were positive for TTC during the second sampling as compared to the first (Figure 3a). Dug wells were consistently contaminated at both samplings, while there was variability in TTC levels between two samples from the same RWH and between two samples from the same spring (Figure 3b). When comparing two samples tested from the same water source, median TTC levels were higher when samples were tested from springs and rainwater systems after rainfall, although these differences were only significant for springs (*p* = 0.038, Wilcox rank sum) (Figure 3c).

## 4. Discussion

Overall, levels of contamination were high: all samples from dug wells contained fecal indicator bacteria, while 95% of springs and 61% of rainwater harvesting systems were contaminated. We did not find significant correlations between TTC counts and sanitary risk scores. Contamination was instead more closely correlated with the water source type (i.e., dug wells were significantly more contaminated than springs, which were significantly more contaminated than rainwater harvesting tanks). RWH were of better quality than dug wells or springs; in our version of the modified sanitary survey, there were fewer possible risk factors for RWH and, by extension, fewer contamination pathways. We found variability in levels of TTC between the two samples tested from the same water source on two different days or before or after a rainfall event, although results did not vary in aggregate.

One risk factor often used as a proxy for safe water is whether a dug well or spring is protected (the Joint Monitoring Program of the WHO/UNICEF, which monitors global access to water, had previously defined an improved dug well or spring as whether these sources were protected [21]). In protected springs, the eye of the spring is enclosed in a covered concrete box with an outlet near the bottom to allow the flow of water away from the original site of the spring. In our sample, all but one spring lacked a surface diversion ditch (which would intercept surface water runoff carrying possible contaminants) or lacked a fence (preventing livestock from accessing the uphill area of the spring). Similarly, wells considered by community members as protected often lacked lining or a windlass. Therefore, while we did not find significant differences in TTC concentrations between protected and unprotected springs and wells, inadequacies in protection may have left these water sources vulnerable to contamination. We also noted that many communities identified these springs and dug wells as protected despite these faults. This highlights the need for communication with communities and water source owners on the proper protection of water sources.

Our study focused on water quality at the point of collection. However, during sampling, we observed that some rainwater harvesting tanks may have included a mix of rainwater and groundwater, a practice used to ensure the availability of water and documented in other studies [22]. This and other water collection and handling-related practices would be expected to further deteriorate water quality [23]. There is a need to extend the safe management of water past the water source and to collection, handling, and storage of water.

While we found that sanitary surveys were not a substitute for microbial water quality testing results in this context, they did identify many faults in the water sources, particularly in sources considered by communities to be protected. Sanitary surveys could be a useful tool for highlighting hazards to address as part of a comprehensive risk management approach system, such as Water Safety Plans [24]. For example, sanitary surveys could be conducted as an initial assessment of water sources to identify systemic hazards in an area (e.g., the sanitary surveys in this study revealed that most wells and springs were unfenced, and that most wells were unsealed). Collective data on sanitary risks could help highlight potential key investments. However, for these to be effective tools, their results should be linked to actions to improve the water sources.

In this study, we expanded on the WHO GDWQ sanitary survey. We included water source infrastructure features, which vary little over time (e.g., lack of fencing or diversion ditch), and potential sources of contamination, which could be persistent (e.g., dirty environment, vegetation near the source) or transient (e.g., animals grazing, clothes washing, human activity). Future research could compare the influence on water quality of blocking pathways to contamination through the water source infrastructure to that of controlling sources of contamination. Transient sources of contamination are particularly challenging to study: several of our sanitary survey questions relied on observations at the time of the survey (e.g., observations of children playing near the source). These observations may have differed if the water source had been sampled at a different time of day. Additionally, sanitary risk scores weighed all risks equally; however, it is likely that some factors may be more influential than others (for example, factors related to the structural integrity of the water source could be more influential than transient sources of pollution such as children playing near the source). Future research should focus on weighting the individual elements of a sanitary survey inspection to identify whether some elements have a higher contribution to risk. Such weighting could use an expert opinion or tools such as decision trees or system dynamics approaches to understand the interactions between hazards [25,26,27].

Finally, the approach used in this study relied on an inventory of water sources to understand where water sources were distributed; we then relied on the knowledge of the Community Health Workers to select from those sources most in use by communities. Water point mapping, a tool frequently used to create inventories of water sources with geo-tagged information, could also serve as useful sampling frames for future studies [28,29,30].

There are several other limitations to our study. First, the study focused on a small set of sub-counties; therefore, the results obtained are specific to the study area and should not be directly applied elsewhere. Second, the number of sources sampled and the total number of samples were limited. It is possible, due to the small sample size, that a small but significant relationship between sanitary risk scores and TTC existed but that the sample size was not sufficient to detect the association. Finally, there is likely further variation in water quality in other seasons. A review of the seasonal effects of fecal contamination in drinking water sources found that improved sources were more contaminated during wet seasons than dry seasons across all water source types and in various climates [30]. While our results showed variation in the water quality of individual sources before or after rainfall events, the study took place during only two months. Cross-sectional studies can potentially underestimate contamination due to seasonal variability in fecal contamination [12,31]; therefore, it would be important for future studies to sample longitudinally, particularly as effects of the identified hazards may be mediated by rainfall.

## 5. Conclusions

We found that sanitary survey results were not associated with microbial water quality testing results in Kisii County, Kenya, although contamination by TTC was associated with source type (dug wells and springs were more contaminated than rainwater systems). We also found variations in water quality between samples tested from the same source at different times, emphasizing the value of using multiple sources of information about risks to water quality. Therefore, while sanitary surveys cannot be substituted for microbial water quality results in this context, they can be used to identify potential hazards and contribute to a comprehensive risk management approach.

## Figures and Tables

**Table 1:** Sanitary survey questions for springs, wells (covered dug well with hand-pump, dug well with windlass and partial cover, and open well), and rainwater adapted from the WHO Guidelines for Drinking Water Quality [8]. The factor present poses a risk; the factor not present means that risk factor does not exist.

**Dug Wells (Including Open Wells and Those with A Handpump) ^b^**		
1	Unprotected by masonry	Is the well unprotected by masonry or concrete wall?
2	Latrine	Is there a latrine <10 m of the well? ^a^
3	Lack cover	Does the well have a cover?
4	Nearest latrine higher	Is the nearest latrine on higher ground than the well? ^a^
5	Pollution	Are there any other source of pollution (e.g., animal excreta, rubbish) <10 m of the well? ^a^
6	Stagnant water	Is there stagnant water <2 m of the well? ^a^
7	Inadequate parapet	Is the wall (parapet) around the well inadequate, allowing surface water to enter the well? ^a^
8	Floor < 1 m	Is the concrete floor <1 m wide around the well (applicable for protected wells)? ^a^
9	Walls unsealed	Are the walls of the well inadequately sealed at any point for 3 m below ground? ^a^
10	Cracks	Are there any cracks in the concrete floor around the well that could permit water to enter the well? ^a^
11	Rope and bucket	Are the rope and bucket left in such a position that they may become contaminated? ^a^
12	Unfenced	Does the installation lack fencing? ^a^
13	Animals grazing	Were animals grazing around the well <2 m at the time of visit?
14	Clothes washing	Were people washing clothes <2 m around the well at the time of visit?
15	Open defecation	Is there open defecation uphill of the site <2 m?
16	Flooding	Is the site unprotected against flooding (located in a depression or along storm water pathway)?
17	Dirty environment	Is the environment around the well dirty?
**Springs**		
1	Unprotected	Is the spring source unprotected by masonry or concrete wall or spring box and therefore open to surface contamination? ^a^
2	Masonry faulty	Is the masonry protecting the spring source faulty? ^a^
3	Unfenced	Is the area around the spring unfenced? ^a^
4	Animals access	Can animals have access to within 10 m of the spring source? ^a^
5	Lack diversion ditch	Does the spring lack a surface water diversion ditch above it, or (if present) is it nonfunctional? ^a^
6	Immediate latrine uphill	Are there any latrines uphill of the spring? ^a^
7	Nearest visible latrine higher	Is the nearest latrine on higher ground than the well?
8	Pollution	Are there any other source of pollution (e.g., animal excreta, rubbish) within 10 m of the well?
9	Animals grazing	Are animals grazing <2 m arround the spring?
10	Clothes washing	Are people washing clothes <2 m uphill of the spring?
11	Open defecation	Is there open defecation uphill the site?
12	Human activity	Are children playing arround the spring?
13	Ponding	Is the spring collection area not developed to minimize ponding of surface water?
14	Vegetation	Is the spring a collection area with deep-rooted vegetation?
**Rainwater Harvesting Systems (RWH)**		
1	Roof contamination	Is there any visible contamination of the roof catchment area (plants, dirt, or excreta)? ^a^
2	Dirty gutters	Are the guttering channels that collect water dirty? ^a^
3	Lack cover	Is there any other point of entry to the tank that is not properly covered? ^a^
4	Defective tap	Is the tap leaking or otherwise defective? ^a^
5	Drainage	Is the water collection area inadequately drained? ^a^
6	Pollution	Is there any source of pollution around the tank or water collection area (e.g., excreta)?
7	Roof type	Is the type of roof thatched (instead of tarred)?
8	Tank opening	Is the opening to the tank not covered?/Is the access hatch not sealed to prevent entry of contaminants?

^a^ Questions that were part of the original WHO sanitary survey; ^b^ Questions were drawn from several WHO sanitary surveys for groundwater sources (open dug well, dug well with windlass and partial cover, covered dug well with hand-pump, covered dug well).

**Table 2:** Water quality in samples from each source type (dug wells, springs, and rainwater harvesting systems (RWH)), including sample size (n), the percent of water samples positive (% pos) for thermotolerant coliform (TTC) and their median (med) concentration, and the median (med), minimum and maximum (range) of total dissolved solids (TDS), turbidity, pH, and temperature.

Water Source	n	TTC (CFU/100 mL)			TDS (mg/L)		Turbidity (NTU)		pH		Temperature (°C)	
		% Pos	Med	Mean (sd) ^a^	Med	Range	Med	Range	Med	Range	Med	Range
Dug wells	34	100%	265	157 (3)	46	6–302	10.8	2.1–173.0	5.7	5–6.31	22.9	17.7–26.7
Springs	41	95%	83	0 (-)	44	23–122	5.7	0.9–49.3	5.7	5.2–6.51	23.4	18.8–28.8
RWH	31	61%	2	0 (-)	6	2–15	3.8	2–7.6	6.4	5.4–7.17	22.1	10.4–26.1
All sources	106 ^b^	87%	107	23 (1)	38	2–303	5.2	0.9–173.0	5.8	5–7.17	23	10.4–28.8

^a^ geometric mean and geometric standard deviation; ^b^ The second samples from 16 sources could not be tested due to problems with the laboratory.

**Table 3:** Individual sanitary risk factors by water source type.

	Dug Wells			Springs			Rainwater Harvesting		
	Sanitary Risk	Percent of Sources (n = 20)	*p* ^a^	Sanitary Risk	Percent of Sources (n = 25)	*p* ^a^	Sanitary Risk	Percent of Sources (n = 16)	*p* ^a^
1	Unprotected by masonry	50%	0.88	Unprotected	44%	0.07	Roof contamination	62%	0.70
2	Latrine nearby	80%	0.29	Masonry faulty	7% ^b^	0.78	Dirty gutters	6%	0.28
3	Lack cover	60%	0.81	Unfenced	88%	0.28	Lack cover	75%	0.71
4	Nearest latrine higher	60%	0.64	Animals access	48%	0.51	Defective tap	19%	0.64
5	Pollution	70%	0.23	Lack diversion ditch	96%	0.68	Drainage	38%	0.25
6	Stagnant water	60%	0.33	Latrines uphill	40%	0.66	Pollution	0%	-
7	Inadequate parapet	70%	0.77	Nearest latrine higher	28%	0.22	Roof type	0%	-
8	Floor < 1 m	0% ^b^	-	Pollution	24%	0.07	Tank opening	56%	0.46
9	Walls unsealed	95%	0.29	Animals grazing	8%	0.96			
10	Cracks	10% ^b^	0.72	Clothes washing	36%	0.44			
11	Rope and bucket	84% ^c^	0.5	Open defecation	4%	0.68			
12	Unfenced	100%	-	Human activity	84%	0.15			
13	Animals grazing	40%	0.72	Ponding	43% ^b^	1			
14	Clothes washing	5%	1	Vegetation	58% ^d^	0.58			
15	Open defecation	5%	0.25						
16	Flooding	60%	0.61						
17	Dirty environment	75%	0.57						
Mean risk score:		58%			45%			32%	

^a^ Wilcox rank sum test for difference between mean TTC concentration in groups with/without sanitary risk factor; ^b^ unprotected sources did not have these features (n = 10 for dug wells and n = 14 for springs); ^c^ n = 19, one was a hand-pump; ^d^ n = 24, as one observation was missing.
